# Efficacy and safety of hepatic arterial infusion chemotherapy combined with fruquintinib and tislelizumab for patients with microsatellite stable colorectal cancer liver metastasis following failure of multiple-line therapy

**DOI:** 10.3389/fonc.2024.1420956

**Published:** 2024-08-20

**Authors:** Kanglian Zheng, Xu Zhu, Liang Xu, Guang Cao, Chuanxin Niu, Xiaoluan Yan, Da Xu, Wei Liu, Quan Bao, Lijun Wang, Kun Wang, Baocai Xing, Xiaodong Wang

**Affiliations:** ^1^ Key Laboratory of Carcinogenesis and Translational Research (Ministry of Education/Beijing), Department of Interventional Therapy, Peking University Cancer Hospital & Institute, Beijing, China; ^2^ Key Laboratory of Carcinogenesis and Translational Research (Ministry of Education/Beijing), Department of Hepatic & Biliary Surgery, Peking University Cancer Hospital & Institute, Beijing, China

**Keywords:** colorectal cancer liver metastasis, microsatellite-stable, hepatic arterial infusion chemotherapy, fruquintinib, tislelizumab

## Abstract

**Background and aim:**

The prognosis of microsatellite stable (MSS)-colorectal cancer liver metastasis (CRCLM) following failure of multi-line therapy remains dismal. The aim of this study is to evaluate the efficacy and safety of hepatic arterial infusion chemotherapy (HAIC) plus fruquintinib and tislelizumab (HAIC-F-T treatment) for MSS-CRCLM which failed from multiple-line therapy.

**Methods:**

From February 2021 to June 2023, 45 patients with MSS-CRCLM after failure of multiple-line therapy who received HAIC combined with fruquintinib and tislelizumab (HAIC-F-T triple treatment) were enrolled. The combination therapy included HAIC regimens with oxaliplatin and 5-fluorouracil or irinotecan, oxaliplatin, and 5-fluorouracil on days 1-2, intravenous tislelizumab (200 mg) before HAIC on day 1, and oral fruquintinb (3 mg/d) on day 3-21, every 4 weeks. Overall survival (OS) and progression-free survival (PFS) were estimated using the Kaplan-Meier method.

**Results:**

The follow-up ended on June 22, 2024, with a median follow-up time of 17.5 months. The objective response rate was 42.2%, and the disease control rate was 82.2%. The median OS was 15.3 months (95% confidence interval [CI]:12.634-17.966), and the median PFS was 7.5 months (95% CI:5.318-9.682). The independent risk factors related to worse OS were previous PD-1 immunotherapy (*P* = 0.021) and the number of HAIC-F-T triple treatment cycles of ≤ 2 (*P* = 0.007). The incidence of grade 3 or higher adverse events (AEs) was 20%, with the most frequent grade 3 or higher AEs being abdominal pain (3/45, 6.7%).

**Conclusion:**

HAIC combined with fruquintinib and tislelizumab may be an alternative salvage treatment for patients with MSS-CRCLM following failure of multiple-line therapy.

## Introduction

Colorectal cancer (CRC) is the third most common malignancy and the second leading cause of cancer-related death worldwide (1). Approximately 20% of patients with CRC have synchronous liver metastasis, and approximately 50% of patients with CRC will develop liver metastasis (2, 3). Liver resection has been confirmed to significantly improve the prognosis of patients with colorectal cancer liver metastasis (CRCLM), whereas only 20% of patients with CRCLM are suitable for liver resection (4, 5).

For patients with unresectable metastatic CRC, systemic chemotherapy with or without targeted therapy (anti-vascular endothelial growth factor [VEGF] or anti-epidermal growth factor receptor [EGFR] therapy) has been demonstrated to prolong survival and is recommended as first- and second-line treatment for metastatic CRC. However, the prognosis of metastatic CRC that has failed standard second-line treatment remains dismal. Although regorafenib, TAS-102, and fruquintinib are recommended as third-line treatments, the median progression-free survival (PFS) and median overall survival (OS) ranged from 1.9 to 3.7 months and from 6.4 to 9.3 months, respectively, in multiple phase III trials (6–10).

Programmed death receptor 1 (PD-1)/programmed death receptor ligand-1 (PD-L1) inhibitors have shown great efficacy in CRC with microsatellite instability-high (MSI-H)/deficiency mismatch repair (dMMR) in some proof-of-concept studies and phase II trials, with an objective response rate (ORR) ranging from 31.1% to 65% (11–14). Nevertheless, in the KEYNOTE-016 and KEYNOTE-028 trial, the efficacy of PD-1/PD-L1 inhibitors for CRC with microsatellite-stable (MSS)/proficiency mismatch repair (pMMR), which accounts for 80-90% of patients with CRC, was dismal, with an ORR of 0% (11, 15, 16). This may due to the absent or inadequate T cell infiltration and an immunosuppressive tumor microenvironment presented by MSS tumor, which leads to the resistance to PD-1/PD-L1 inhibitors (17). However, anti-VEGF therapy and PD-1/PD-L1 inhibitors presented with synergistic effect in treating MSS-CRC in recent years, with an ORR ranging from 7.1% to 60% (18–20). Fruquintinib, an oral multi-kinase inhibitor targeting VEGF receptors 1-3, was recently demonstrated to enhance the efficacy of PD-1/PD-L1 inhibitors for MSS-CRC (21, 22). A median PFS ranging from 3.4 to 5.6 months was achieved after treatment of the combination of fruquintinib and PD-1/PD-L1 inhibitors in MSS-CRCLM in preliminary studies (23, 24).

Hepatic arterial infusion chemotherapy (HAIC) can deliver chemotherapeutic agents directly into the vessels supplying the tumor in the liver, reaching a high concentration of chemotherapeutic agents in the tumor and achieving great local tumor control in the liver (25). As HAIC has been shown to be beneficial to unresectable CRCLM for many years, it is recommended as a salvage treatment following failure of standard systemic treatment (26–29). In 2021, a meta-analysis showed that both the OS rate and ORR in the HAIC group were significantly higher than those in the systemic therapy group (30). The hazard ratio of the OS rate was 0.17 (*P* < 0.001) in the palliative treatment setting and 0.63 (*P* < 0.001) in the adjuvant setting, and the relative risk of ORR was 2.09 (*P* = 0.001) in the palliative treatment setting and 2.14 (*P* < 0.001) in the adjuvant setting.

Thus, this retrospective study aimed to evaluate the efficacy and safety of HAIC combined with fruquintinib and tislelizumab (a PD-1 inhibitor) for patients with MSS-CRCLM following failure of multiple-line therapy.

## Materials and methods

### Patients

This retrospective study was approved by the ethics committee of the Peking University Cancer Hospital, and the need for informed consent was waived. The study was performed in line with the principles of the Declaration of Helsinki. All patients with CRCLM who were treated by HAIC combined with fruquintinib and tislelizumab from February 2021 to June 2023 were reviewed.

The inclusion criteria were as follows: (1) 18-80 years old; (2) diagnosed with unresectable CRCLM by histopathology and confirmed by a multidisciplinary team; (3) after failure of multiple-line therapy; (4) at least one measurable lesion in the liver according to the RECIST 1.1 criteria; (5) microsatellite stable; and (6) at least one evaluation of the tumor response to the treatment. Patients with other concomitant malignancies were excluded, as were those with a lack of baseline or follow-up data and a follow-up time of < 6 months.

**Figure 1:**
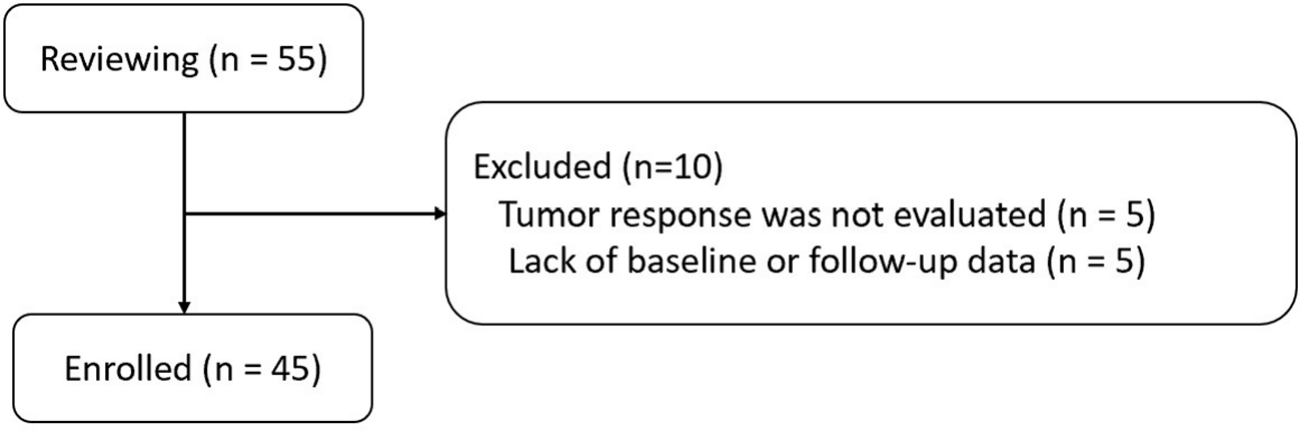
Flowchart of patient enrollment.

Searching for patients in the Hospital Information System-based Case Retrieval System via the keywords “CRCLM,” “fruquintinib,” and “tislelizumab,” 55 patients with CRCLM treated by HAIC combined with fruquintinib and tislelizumab were identified for the period from February 2021 to June 2023. Finally, 45 patients with MSS-CRCLM were enrolled in this study according to the inclusion and exclusion criteria (**Figure 1**).

### Procedures and treatment regimens

All patients underwent abdominal contrast-enhanced CT/MRI within 1 month before the initiation of the treatment, and blood tests, such as blood routine examination, blood biochemistry, and tumor markers, were performed within 3 days before the initiation of the treatment.

The treatment, which was repeated every 4 weeks, consisted of HAIC, fruquintinib, and tislelizumab (HAIC-F-T triple treatment). The HAIC regimens, which depended on the decision of clinicians based on the previous regimens of systemic chemotherapy, were oxaliplatin (85 mg/m^2^, 0-2h, split into d1 and d2) and 5-fluorouracil (2 g/m^2^, 2-24h, split into d1 and d2) or irinotecan (100 mg/m2, 0-2 h, d1), oxaliplatin (65 mg/m2, 0-2 h, d2) and 5-fluorouracial (2 g/m2, 2-24 h, split into d1 and d2). Fruquintinib was administered orally after HAIC at a dosage of 3 mg on days 3-23, then suspended for 1 week. Tislelizumab was intravenously administered at a dosage of 200 mg before 24 h of HAIC.

HAIC was performed via a temporary indwelling hepatic artery catheter inserted as follows: After puncturing the femoral artery using the Seldinger technique, celiac and superior mesenteric angiographies were performed to detect variations of the hepatic artery. As described in previous published study, extrahepatic blood flow redistribution was performed via a 2.4/2.7F microcatheter to embolize the arteries that supply the extrahepatic organs, such as right gastric artery and accessory left gastric artery, with micro-coils; and intrahepatic blood flow redistribution were performed to convert the multiple hepatic arteries into one hepatic artery in cases of multiple hepatic artery variations such as accessory right/left hepatic artery arising from superior mesenteric artery or left gastric artery (31). The micro-catheter was then placed in the proper hepatic artery or common hepatic artery to ensure whole-liver perfusion via HAIC. HAIC was performed in the ward, and the catheter and sheath were removed following its completion. The same procedure was repeated for the next cycle of HAIC.

The combination of fruquintinib and tislelizumab was administered as maintenance treatment until tumor progression or patients died for patients who achieved liver tumor control after four to six cycles of the HAIC-F-T triple treatment.

### Assessment and follow-up

Blood tests, such as blood routine examination, blood biochemistry, and tumor markers, were performed before every cycle of the treatment or every 3 months until tumor progression or death of patients, while abdominal contrast-enhanced CT/MRI was performed after every two cycles or every 3 months until tumor progression or death of patients. The CT/MRI images were analyzed by two radiologists with 12 and 17 years of experience in diagnostic imaging. Liver tumor burden was defined as the extent of liver metastasis proportional to the whole liver volume before the initiation of the combination therapy, and liver metastasis dominant was defined as a proportion of liver metastasis to systemic metastasis of ≥ 75% before the initiation of the combination treatment. The liver tumor burden and liver metastasis, irrespective of whether it was dominant or not, were independently assessed by radiologists, and a consensus was obtained.

Tumor response was evaluated using the RECIST 1.1 criteria. The ORR consisted of the complete response (CR) and partial response (PR), and the disease control rate (DCR) consisted of CR, PR, and stable disease (SD). Treatment related adverse events (AEs) were assessed using the National Cancer Institute-Common Terminology Criteria for Adverse Events (NCI-CTCAE) Version 5.0.

### Statistical analysis

Continuous variables are described as the mean ± standard deviation (SD), and were analyzed using chi-square tests or Fisher’s tests. Categorical variables are described as proportions, which were analyzed using Wilcoxon signed-rank tests. The OS and PFS were calculated using the Kaplan-Meier method and assessed using log-rank tests. The OS was calculated from the initiation of HAIC-F-T triple treatment to the time of the patients’ death or last follow-up, and the PFS was calculated from the initiation of HAIC-F-T triple treatment to the time of disease progression or patients’ death, whichever occurred first. Univariate and multivariate analyses were performed using the Cox proportional hazards regression method to detect risk factors related to worse survival, and characteristics with *P* < 0.1 in univariate analysis were included in the multivariate analysis. *P* < 0.05 was considered as statistically significant. The optimal cut-off values for continuous variables, such as the CEA level, CA 19-9 level, and time from liver metastasis to HAIC-F-T triple treatment, were determined using X-tile software (version 3.6.1, Yale University, New Haven, CT, USA). All other statistical analyses were performed using R software (R version 4.2.0, http://www.r-project.org).

## Results

### Patients

Of the 45 patients with MSS-CRCLM finally who were enrolled in this retrospective study, the median age was 58.73 ± 7.86 years, and 23 (51.1%) were male. Twenty-six patients (57.8%) were diagnosed with left colon cancer, 8 (17.8%) with right colon cancer, and 11 (24.4%) with rectal cancer. Most patients (73.3%) had primary tumors resected, and 82.2% had extrahepatic metastases. All patients had previously received second- or more-line oxaliplatin-based or irinotecan-based doublet or triplet chemotherapy with targeted treatment before HAIC-F-T triple treatment. Twenty-five patients (55.6%) had received more than three-line previous treatment, and 29 patients (64.4%) had received previous HAIC, with a mean of 3.06 ± 2.58 cycles, while got progression before HAIC-F-T triple treatment. The characteristics of the patients are detailed in **Table 1**.

**Table 1:** Patients’ characteristics.

Characteristics	N (%)	Characteristics	N (%)
Gender		Previous treatment line	
Male	23 (51.1)	≤ 3	20 (44.4)
Female	22 (48.9)	> 3	25 (55.6)
Age (y)		Previous PD-1 immunotherapy	
≤ 60	24 (53.3)	Yes	8 (17.8)
> 60	21 (46.7)	No	37 (82.2)
Primary tumor site		Previous triplet chemotherapy	
Left colon	26 (57.8)	Yes	23 (51.1)
Right colon	8 (17.8)	No	22 (48.9)
Rectum	11 (24.4)	Previous HAIC within 6 months before HAIC-F-T triple treatment	
Primary tumor resected		Yes	23 (51.1)
No	12 (26.7)	No	22 (48.9)
Yes	33 (73.3)	Extrahepatic metastasis	
RAS genotype		Yes	37 (82.2)
Wild type	17 (37.8)	No	8 (17.8)
Mutant type	28 (62.2)	Liver tumor burden	
Differentiation		≤ 50%	27 (60)
Well	1 (2.3)	> 50%	18 (40)
Moderate	36 (80)	Liver metastasis dominant	
Poor	6 (13.3)	No	15 (33.3)
Unknown	2 (4.4)	Yes	30 (66.7)
ECOG		Number of liver metastasis	
0	29 (64.4)	< 10	23 (51.1)
1	16 (35.6)	≥ 10	22 (48.9)
Child-Pugh classification		Time from liver metastasis to initiation of HAIC-F-T triple treatment	
A	42 (93.3)	≤ 21.8 months	25 (55.6)
B	3 (6.7)	> 21.8 months	20 (44.4)
CEA level		Number of HAIC-F-T triple treatment	
≤ 1771.2 ng/ml	37 (82.2)	≤ 2 cycles	12 (26.7)
> 1771.2 ng/ml	8 (17.8)	> 2 cycles	33 (73.3)
CA 19-9 level		Combined with TACE	
≤ 606.4 U/ml	25 (55.6)	Yes	16 (35.6)
> 606.4 U/ml	18 (40)	No	29 (64.4)
Unknown	2 (4.4)	Regimen of HAIC	
Liver metastasis		Doublet regimen	30 (66.7)
Synchronous	36 (80)	Triplet regimen	15 (33.3)
Metachronous	9 (20)		

ECOG, Eastern Cooperation Oncology Group; CEA, carcinoembryonic antigen; CA 19-9, carbohydrate antigen 19-9; PD-1, programmed death receptor 1; HAIC, hepatic arterial infusion chemotherapy; TACE, trans-arterial chemoembolization.

### Efficacy

A total of 165 cycles (mean ± SD: 3.7 ± 1.7 cycles) of HAIC-F-T triple treatment were performed in this study. The follow-up procedures were continued until June 22, 2024, with a median follow-up time of 17.5 months.

None of the patients achieved CR in this study, whereas PR, SD, and progressive disease (PD) were achieved in 19 (42.2%), 18 (40%), and 8 (17.8%) patients, respectively, according to the RECIST 1.1 criteria. The ORR was 42.2%, and the DCR was 82.2%.

**Figure 2:**
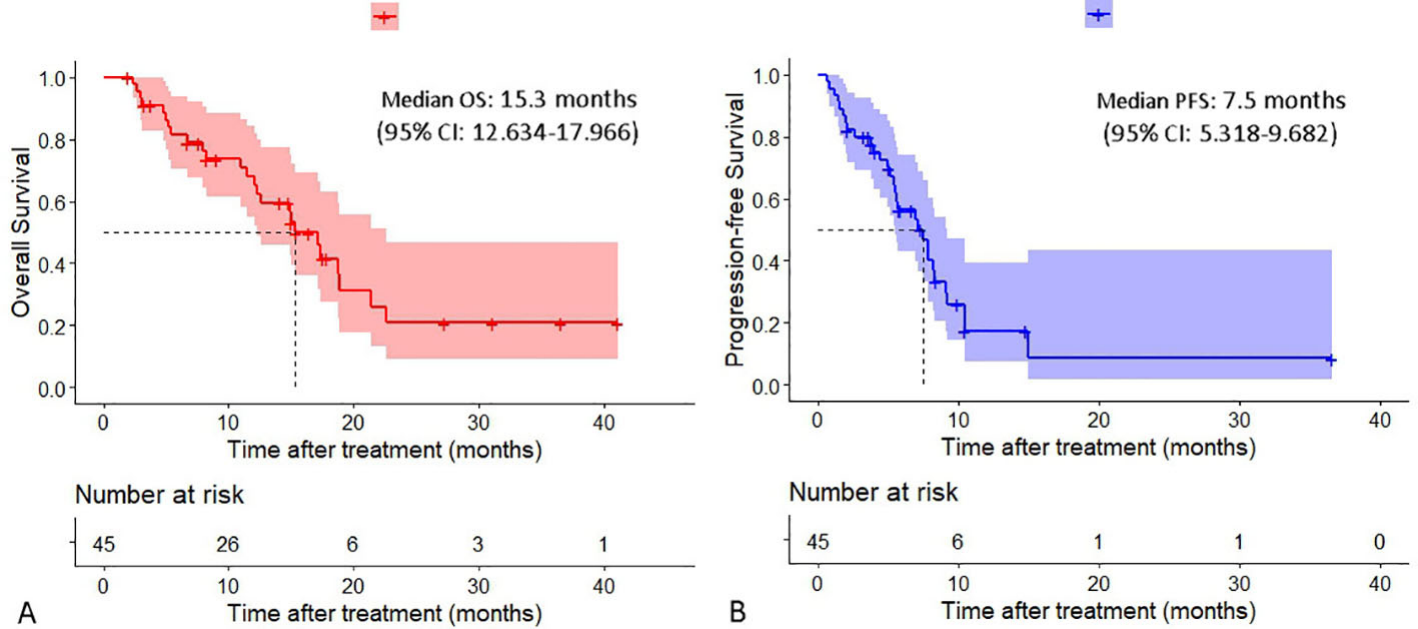
Cumulative curves of overall survival and progression-free survival. **(A)** Overall survival. **(B)** Progression-free survival.

The median OS was 15.3 months (95% confidence interval [CI]:12.634-17.966), and the median PFS was 7.5 months (95% CI:5.318-9.682) (**Figure 2**). The intrahepatic PFS, which was calculated from the initiation of the combination therapy to progression of intrahepatic lesions or patients’ death, whichever occurred first, was 7.9 months (95% CI:6.931-8.869), whereas the extrahepatic PFS, which was calculated from the initiation of the combination therapy to progression of extrahepatic lesions or patients’ death, whichever occurred first, was 5.7 months (95% CI:4.124-7.276).

**Figure 3:**
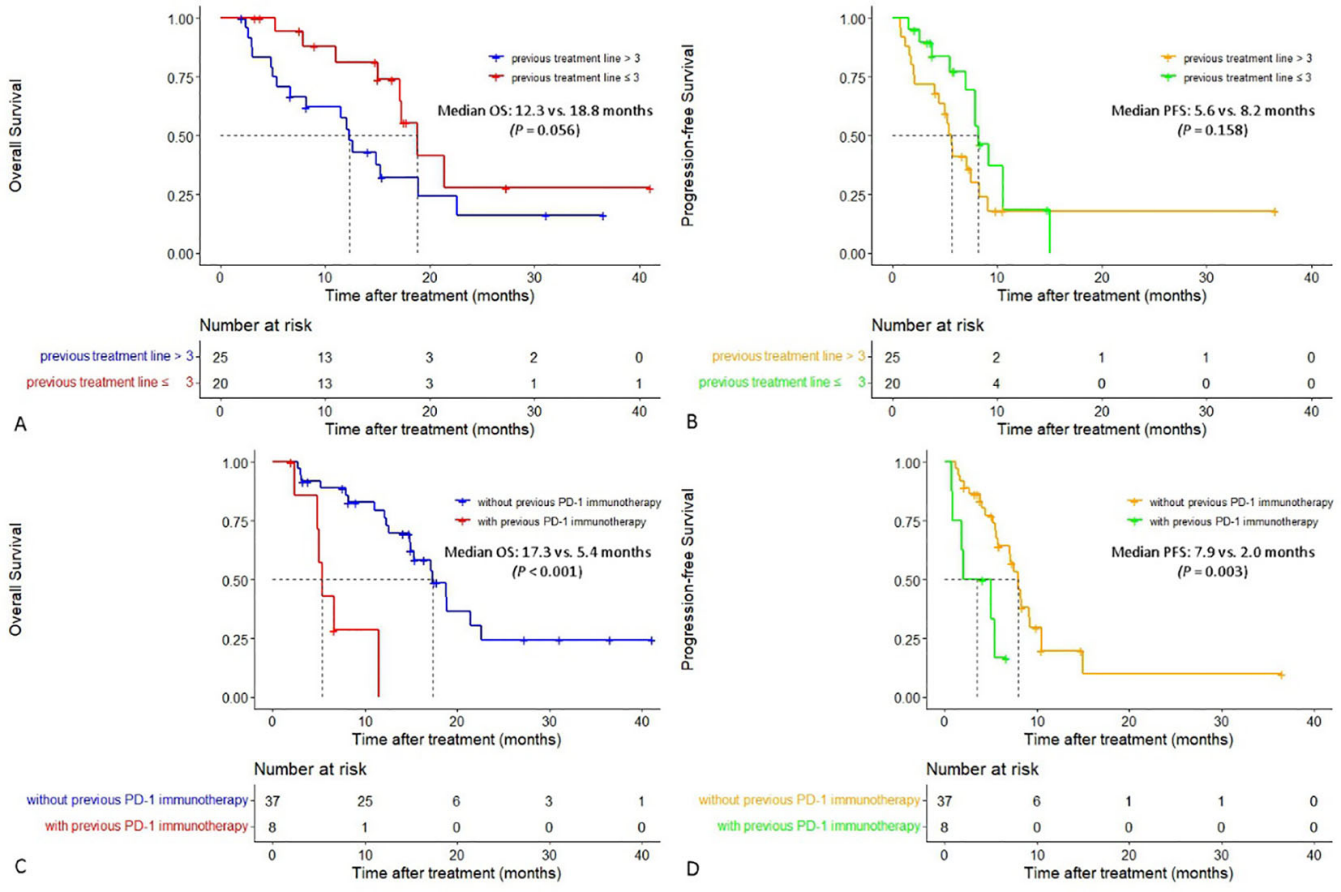
Cumulative curves of overall survival and progression-free survival in different subgroups. **(A, B)** Cumulative curves of overall survival and progression-free survival in patients with different previous treatment lines. **(C, D)** Cumulative curves of overall survival and progression-free survival in patients with or without previous PD-1 immunotherapy.

In patients with two or three lines of previous treatment, the median OS was 18.8 months (95% CI:15.401-22.199), which was longer than that in patients whose previous treatment line was > 3 (12.3 months [95% CI:10.714-13.886], *P* = 0.056), and a tendency for prolonged median PFS was also observed (8.2 vs. 5.6 months, *P* = 0.158). In patients who did not receive previous PD-1 immunotherapy, both the median PFS and OS were significantly longer than those in patients who received previous PD-1 immunotherapy, with a median PFS of 7.9 vs. 2.0 months (*P* = 0.003) and a median OS of 17.3 vs. 5.4 months (*P* < 0.001), respectively (**Figure 3**).

**Figure 4:**
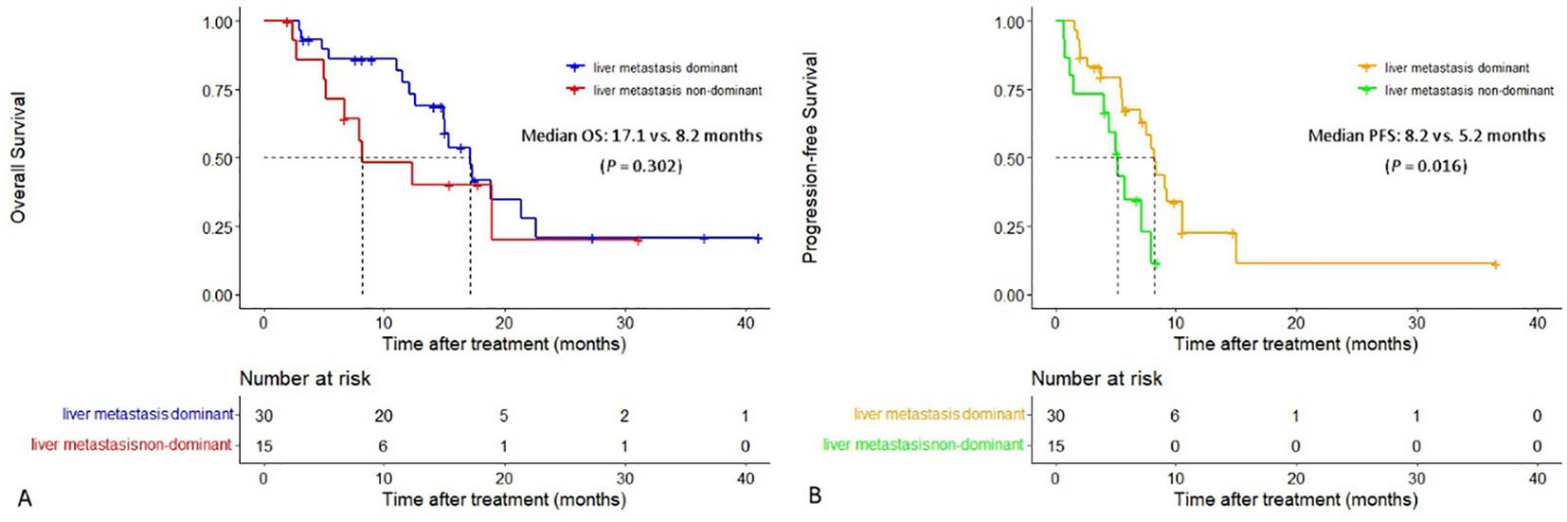
Cumulative curves of overall survival and progression-free survival in patients with liver metastasis dominant and those with liver metastasis non-dominant. **(A)** Overall survival. **(B)** Progression-free survival.

The median PFS in patients with liver metastasis dominant was significantly longer than that in patients with liver metastasis non-dominant (8.2 vs. 5.2 months, *P* = 0.016). The extrahepatic PFS in patients with liver metastasis dominant and patients with liver metastasis non-dominant was 6.6 vs. 5.2 months (*P* = 0.066). Additionally, both median OS and intrahepatic PFS in the former group were longer than that in the latter group (median OS: 17.1 vs. 8.2 months, median intrahepatic PFS: 8.3 vs. 7.1 months), although the differences were not statistically significant (*P* = 0.302 and *P* = 0.100) (**Figure 4**).

### Univariate and multivariate analyses of factors related to OS

In the univariate analysis, CEA > 1771.2 ng/ml (*P* = 0.002), CA 19-9 > 606.4 U/ml (*P* = 0.014), previous treatment line > 3 (*P* = 0.062), previous immunotherapy (*P* < 0.001), and the number of HAIC-F-T triple treatment cycles of ≤ 2 (*P* = 0.002) were found to be risk factors associated with worse OS. After multivariate analysis, previous immunotherapy (*P* = 0.021) and the number of HAIC-F-T triple treatment cycles of ≤ 2 (*P* = 0.007) were identified as independent risk factors related to worse OS (**Table 2**).

**Table 2:** Univariate and Multivariate Analysis of Factors Related to Overall Survival.

Characteristics	Univariate Analysis		Multivariate Analysis	
	HR (95% CI)	*P* value	HR (95% CI)	*P* value
Gender		0.637		
Male	1			
Female	1.209 (0.549-2.663)			
Age (y)		0.349		
≤ 60	1			
> 60	1.460 (0.661-3.228)			
Primary tumor site		0.236		
Left colon	1			
Right colon	1.083 (0.304-3.865)			
Rectum	2.121 (0.879-5.118)			
Primary tumor resected		0.395		
No	1			
Yes	1.592 (0.545-4.653)			
RAS genotype		0.103		
Wild type	1			
Mutant type	2.012 (0.869-4.660)			
Differentiation		0.896		
Well	1			
Moderate	25420.897 (0-5.715E+110)			
Poor	32826.417 (0-7.394E+110)			
ECOG		0.854		
0	1			
1	0.921 (0.383-2.215)			
Child-Pugh classification		0.515		
A	1			
B	1.998 (0.248-16.068)			
CEA level		0.002		0.198
≤ 1771.2 ng/ml	1		1	
> 1771.2 ng/ml	6.029 (1.956-18.581)		2.613 (0.605-11.287)	
CA 19-9 level		0.014		0.079
≤ 606.4 U/ml	1		1	
> 606.4 U/ml	2.840 (1.235-6.533)		3.267 (0.884-12.069)	
Liver metastasis		0.677		
Synchronous	1			
Metachronous	1.218 (0.481-3.082)			
Previous treatment line		0.062		0.071
≤ 3	1		1	
> 3	2.235 (0.959-5.210)		3.248 (0.905-11.660)	
Previous PD-1 immunotherapy		< 0.001		0.021
Yes	1		1	
No	0.119 (0.038-0.373)		0.153 (0.031-0.755)	
Previous triplet chemotherapy		0.535		
Yes	1			
No	0.780 (0.355-1.711)			
Previous HAIC within 6 months before HAIC-F-T triple treatment		0.797		
Yes	1			
No	0.574 (0.363-1.754)			
Extrahepatic metastasis		0.957		
Yes	1			
No	0.926 (0.379-2.415)			
Liver tumor burden		0.538		
≤ 50%	1			
> 50%	1.288 (0.576-2.879)			
Liver metastasis dominant		0.306		
No	1			
Yes	0.651 (0.287-1.479)			
Number of liver metastasis		0.326		
< 10	1			
≥ 10	1.487 (0.673-3.284)			
Time from liver metastasis to initiation of HAIC-F-T triple treatment		0.102		
≤ 21.8 months	1			
> 21.8 months	1.932 (0.878-4.252)			
Number of HAIC-F-T triple treatment		0.002		0.007
≤ 2 cycles	1		1	
> 2 cycles	0.241 (0.098-0.595)		0.174 (0.048-0.624)	
Combined with TACE		0.786		
Yes	1			
No	1.118 (0.499-2.507)			
Regimen of HAIC		0.116		
Doublet regimen	1			
Triplet regimen	0.452 (0.168-1.217)			

ECOG, Eastern Cooperation Oncology Group; CEA, carcinoembryonic antigen; CA 19-9, carbohydrate antigen 19-9; PD-1, programmed death receptor 1; HAIC, hepatic arterial infusion chemotherapy; TACE, trans-arterial chemoembolization.

### Accompanying and subsequent treatment

**Figure 5:**
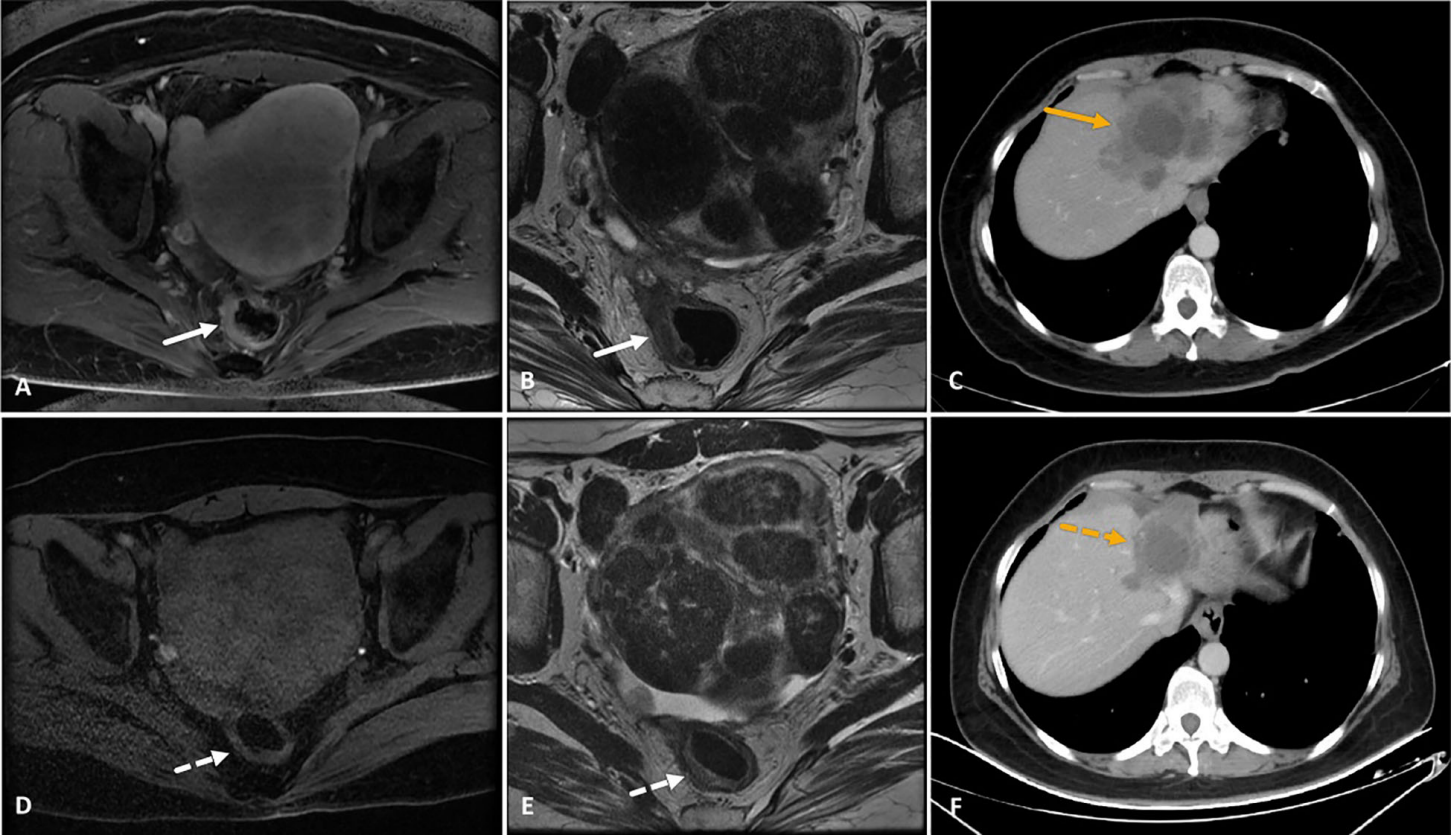
A 39-year-old female patient with microsatellite stable-rectal cancer liver metastasis and RAS wild type who received HAIC (irinotecan, oxaliplatin, and 5-fluorouracil) combined with fruquintinib and tislelizumab as third-line treatment. **(A–C)** MRI images showed a thickened rectal wall with significant enhancement (white arrows in images **A** and **B**), and CT image showed progress of liver metastasis (orange arrow in image **C**) after two lines of systemic chemotherapy and three cycles of HAIC. **(D–F)** After six consecutive cycles of HAIC-F-T triple treatment, the rectal wall exhibited significant thinning, with a significantly attenuated degree of enhancement (white dotted arrows in images **D** and **E**). Correspondingly, liver metastasis significantly decreased in both size and enhancement degree (orange dotted arrow in image **F**). The tumor response was partial response based on the RECIST 1.1 criteria. The patient underwent partial liver resection and radical rectal cancer resection subsequently, and histopathological examination showed a pathological complete response in both primary tumor and liver metastasis. Although PET-CT detected active lymph nodes in the retroperitoneal space 8.4 months after the surgery, this was relieved after subsequent radiation therapy. Up to the last follow-up, the patient was still alive, with an overall survival of 41.1 months after the HAIC-F-T triple treatment.

Drug-eluting trans-arterial chemoembolization was performed in 16 patients (35.6%) who had multiple lesions in the bi-lobe of the liver, with a mean of 1.2 ± 2.7 cycles before HAIC treatment. Among the five patients who achieved successful liver metastasis downstaging conversion, two (4.4%) received ablation and two (4.4%) received radiation therapy. Another patient underwent surgical resection after the HAIC-F-T triple treatment, and histopathological examination showed a pathological complete response in both primary tumor and liver metastasis (**Figure 5**). After tumor progression, 12 patients (26.7%) received HAIC with other regimens, 17 patients (37.8%) received systemic chemotherapy, and 9 patients (20%) received regorafenib, based on the progression of intrahepatic or extrahepatic metastasis.

### Safety

No treatment-related deaths occurred in this study, and none of the patients had catheter-related complications during HAIC. Treatment-related AEs, which occurred in all patients, were manageable, and most of them had recovered to normal before the next cycle of study treatment without any medical intervention. Grade 3 or higher AEs were observed in only 9 patients (20%), and the most frequent grade 3 or higher AEs were abdominal pain (3/45, 6.7%) and hand-foot syndrome (2/45, 4.4%). One patient suspended the administration of fruquintinib because of grade 3 proteinuria, while got progressed because of extrahepatic metastasis during the suspension. All treatment-related AEs are detailed in **Table 3**.

**Table 3:** Treatment-related Adverse Events.

Adverse Events	Any grade (N [%])	Grade 3 or higher (N [%])
Hematological toxicity		
Leukopenia	24 (53.3)	1 (2.2)
Neutropenia	10 (22.2)	0
Thrombocytopenia	20 (44.4)	1 (2.2)
Anemia	16 (35.6)	0
Non-hematological toxicity		
Elevated ALT/AST	28 (62.2)	0
Elevated total bilirubin	19 (42.2)	0
Abdominal pain	16 (35.6)	3 (6.7)
Diarrhea	4 (4.4)	0
Nausea	32 (71.1)	0
Vomiting	17 (37.8)	1 (2.2)
Fatigue	9 (20)	0
Hand-foot syndrome	7 (15.6)	2 (4.4)
Neurological toxicity	5 (11.1)	0
Hypertension	5 (11.1)	1 (2.2)
Rash	9 (20)	0
Proteinuria	1 (2.2)	1 (2.2)
Epistaxis	5 (11.1)	0
Immunotherapy-related toxicity		
Hypothyroidism	1 (2.2)	0
Pneumonia	1 (2.2)	1 (2.2)

ALT, alanine transaminase; AST, aspartate transaminase.

Immunotherapy-related AEs were observed in two patients (4.4%). Hypothyroidism was detected in one patient after two cycles of the study treatment, and levothyroxine was administered without suspension or interruption of tislelizumab administration. However, another patient was diagnosed with immunotherapy-related pneumonia after two cycles of the HAIC-F-T triple treatment, which required the interruption of tislelizumab administration, hospitalization for best supportive care, and the administration of adrenocortical hormone agents.

## Discussion

The survival benefits of the recommended third-line treatment remain limited, with a median PFS of up to 3.7 months and a median OS of up to 9.3 months (6–10). In the present study, HAIC combined with fruquintinib and tislelizumab (HAIC-F-T triple treatment) showed great efficacy in patients with MSS-CRCLM following failure of standard second- or more-line therapies. The ORR was 42.2%, and the DCR was 82.2%, with a median OS and median PFS of 15.3 and 7.5 months, respectively, all of which were better than the standard third-line treatment of CRCLM.

Fruquintinb is a multi-kinase inhibitor that targets at VEGF receptors 1-3. Recently, some preclinical studies showed that fruquintinib could enhance the anti-tumor efficacy of PD-1/PD-L1 inhibitors for treating MSS-metastatic CRC by decreasing angiogenesis, reprograming the structure of vessels, and increasing the infiltration of CD8^+^ T cells, CD8^+^TNFα^+^ T cells, and CD8^+^IFNγ^+^ T cells in the tumor microenvironment (21, 22). Additionally, the combination of fruquintinib and PD-1/PD-L1 inhibitors showed great efficacy for patients with MSS-metastatic CRC who failed standard treatment in some retrospective studies and phase II trials, with an ORR, median PFS, and median OS ranging from 7.1% to 21.05%, 5.4 to 9.6 months, and 11.1 to 13.7 months, respectively (18, 23, 24, 32). However, the survival benefits were compromised in patients presenting with liver metastasis, with the median PFS ranging from 3.4 to 5.6 months (23, 24).

HAIC has been explored for the treatment of patients with CRCLM since 1987, and has shown survival benefits as a first-line and adjuvant treatment for patients with CRCLM in some phase III trials (27, 33–36). Recently, HAIC, using double or triple agents, with or without systemic chemotherapy were investigated and showed high local tumor control and survival benefits as a salvage treatment for patients with CRCLM following failure of systemic chemotherapy, with an ORR ranging from 22.4% to 36% and median OS ranging from 13.1 to 32.8 months, respectively (28, 37–39). However, systemic therapy had been demonstrated to relate to greater tumor control for extrahepatic tumor than HAIC (27). Thus, it was suggested to combine HAIC with systemic therapy for treating patients who had both intrahepatic and extrahepatic metastases.

The synergistic effect of the triple combination of HAIC, anti-VEGF, and PD-1 inhibitors involved in this study protocol has been explored previously. A decade ago, anti-VEGF therapy was demonstrated to normalize the vessels and improve their permeability, thus showing a synergistic effect with chemotherapy (40, 41). Meanwhile, chemotherapy has been demonstrated to be synergistic with immunotherapy by directly stimulating the immune system, modulating the immunosuppressive microenvironment, and enhancing immunogenicity (42–45). Recently, the synergistic effects of the triple combination, which includes HAIC, anti-angiogenesis therapy, and immunotherapy for treating malignancies, have been increasingly explored and have shown great efficacy in hepatocellular carcinoma and biliary tract cancer as first-line treatment in some phase II trials (46, 47). In the European Society of Medical Oncology Congress 2023, Wang et al. reported the interim analysis of a phase II trial, which evaluated the efficacy and safety of HAIC combined with fruquintinib and tislelizumab for advanced CRCLM, with an ORR and DCR of 27.59% and 93.1%, respectively; however, neither the median OS nor the median PFS has been reached yet (48). To the best of our knowledge, to date, the present study, which uses this triple regimen for the treatment of CRCLM, is the earliest to start enrolling, with the largest sample size and completed survival data. In the present study, even 64.4% of patients had already received HAIC, 55.6% received more than three lines of treatment before HAIC-F-T triple treatment, and 82.2% had extrahepatic metastasis, the median OS and PFS of 15.3 and 7.5 months, respectively, were achieved, which suggested that the HAIC-F-T triple treatment is a reasonable treatment choice for previous heavily treated patients with CRCLM, even for those previously treated with HAIC and those had extrahepatic metastasis.

Notably, the subgroup analysis showed that the median OS in patients whose previous treatment line 2-3 was longer than that in patients whose previous treatment line was > 3 (18.8 vs. 12.3 months), and both the median PFS and OS in patients who did not receive previous PD-1 immunotherapy were significantly longer than those in patients who received previous PD-1 immunotherapy (median PFS: 7.9 vs. 2.0 months, *P* = 0.003; median OS: 17.3 vs. 5.4 months, *P* < 0.001). The stronger the previous chemotherapy, the more severe the chemotherapy resistance and damage to the immune function of the whole body. The earlier application of HAIC could lead to better local tumor response and the release of more tumor antigens from chemotherapy-induced immunogenic death, thereby producing stronger synergistic therapeutic effects with PD-1 inhibitors. Thus, it has been suggested that the HAIC-F-T triple treatment may be used earlier in patients with advanced MSS-CRCLM. Moreover, multivariate analysis showed that the number of HAIC-F-T triple treatment cycles ≤ 2 was an independent risk factor related to worse OS (*P* = 0.007), indicating that more cycles of HAIC are crucial to the HAIC-F-T triple treatment.

Both the median PFS and OS in patients with liver metastasis dominant were longer than those in patients with liver metastasis non-dominant (8.2 vs. 5.2 months and 17.1 vs. 8.2 months, respectively), indicating the patients with liver metastasis dominant are more likely to benefit from this HAIC-F-T triple treatment. This result may suggest that tumors in patients with liver metastasis non-dominant presented with higher spatiotemporal heterogeneity, which was deemed to be associated with worse prognosis, than tumors in patients with liver metastasis dominant (49). Accordingly, liver metastasis dominant without obvious extrahepatic metastasis is suggested as the optimal indication of this HAIC-F-T triple treatment. Furthermore, it is speculated that immunogenic death of liver metastasis caused by HAIC is insufficient to stimulate a systemic immune response and synergistically inactivate the widespread systemic metastasis for patients with liver metastasis non-dominant.

Regarding the treatment-related AEs in this study, most of treatment-related AEs was grade 1-2, and most had recovered to normal before the initiation of the next combination treatment; however, in one patient who developed immunotherapy-related pneumonia, it was necessary to interrupt the administration of tislelizumab. Notably, only 20% of patients experienced grade 3 or higher AEs in this study. Compared with the CORRECT, CONCUR, RECOURSE, FRESCO, and FRESCO-2 trials, the incidence of grade 3 or higher AEs in this study was more acceptable (20% vs. 54% vs. 61.2% vs. 63% vs. 69%), which may be due to the following reasons: first, the exposure of the whole body to chemotherapeutic agents was less during HAIC due to the first-pass effect in the liver; second, the dosage of fruquintinib administered in this study was less than the standard dosage in the FRESCO and FRECSO-2 trial (6–10).

However, this study has some limitations that warrant discussion. First, the results might be biased due to the retrospective data collection. Second, the sample size was small, with only 45 patients enrolled. Third, 82.2% of patients with extrahepatic metastasis and 33.3% with liver metastasis non-dominant were enrolled in this study, which may have compromised the efficacy of this HAIC-F-T triple treatment. Fourth, the multivariate analysis showed that the number of HAIC-F-T triple treatment cycles of ≤ 2 was one of the independent risk factors related to worse OS (*P* = 0.001); thus, the mean of 3.6 ± 1.6 cycles might limit the optimal survival benefits of the HAIC-F-T triple treatment. At last, this study is preliminary, prospective study is ongoing to confirm the efficacy and safety of the HAIC-F-T triple treatment for MSS-CRCLM.

## Conclusion

HAIC combined with fruquintinib and tislelizumab may serve as an alternative treatment method for MSS-CRCLM that has failed multiple-line therapy, because of its high efficacy and acceptable safety. The number of treatment cycles is closely related to the survival efficacy of this HAIC-F-T triple treatment. The HAIC-F-T triple treatment may be used earlier in patients with MSS-CRCLM and liver metastasis dominant. The results of this study should be verified further in prospective trials with large number of patients with MSS-CRCLM.

## Data Availability

The datasets generated during and/or analyzed during the current study are available from the XW on reasonable request.
